# HbA1c for type 2 diabetes diagnosis in Africans and African Americans: Personalized medicine NOW!

**DOI:** 10.1371/journal.pmed.1002384

**Published:** 2017-09-12

**Authors:** Andrew D. Paterson

**Affiliations:** 1 Genetics and Genome Biology, The Hospital for Sick Children Research Institute, Toronto, Ontario, Canada; 2 Divisions of Epidemiology and Biostatistics, Dalla Lana School of Public Health, University of Toronto, Toronto, Ontario, Canada

## Abstract

Andrew Paterson discusses findings from a new study that shows HbA1c screening for diabetes will leave 2% of African Americans undiagnosed and how personalised medicine is needed.

In the last 10 years, genome-wide association studies (GWASs), in which single nucleotide polymorphisms (SNPs) from across the genome are tested for association with either quantitative traits or disease status, have resulted in the identification of >10,000 novel loci in humans [1,2]. These ultimately provide an unparalleled insight into genes and mechanisms causally related to hundreds of medically important traits and diseases. However, 1 common criticism of the results of GWASs are that they rarely have clinical relevance, mostly due to small effect sizes and the large number of loci.

## A common G6PD variant associated with HbA1c in African Americans without diabetes

The implications of 1 specific finding reported by Wheeler et al. [3], cannot be so easily dismissed. They performed GWASs for HbA1c in people without diabetes (all forms) from multiple ethnic groups and identified a common missense variant, rs1050828 (G202A, p.Val68Met), which contributes with minor allele (T) at nearby rs1050829 to the A^−^ haplotype of Glucose-6-phosphate dehydrogenase, *G6PD* [4], to be associated with lower HbA1c. rs1050828 has a minor allele frequency of 10%–15% in 7,564 African Americans without diabetes from 9 studies, but it is not polymorphic in European or Asian populations used for imputing genotypes. G6PD is on chromosome Xq28, thus, there are 5 possible genotypes at this SNP: African American males hemizygous for the minor allele at this variant (T) have HbA1c 0.79% (8.6 mmol/mol) lower than males without (C), heterozygous females (CT) have HbA1c 0.26% (2.8 mmol/mol) lower, and homozygous females (TT, 1%–2% of women) have HbA1c 0.67% (7.3 mmol/mol) lower than female common homozygotes (CC). Remarkably, in African Americans, the variance in HbA1c explained by the variant is 13%–20% in males and 2%–10% in females, indicating a large effect.

The variant was first identified as a cause of G6PD deficiency in the late 1980’s: different variants have varying effects, and this variant results in moderate deficiency with 10%–60% residual enzyme activity [5]. Importantly, using data from GWASs of glucose in the same African Americans, Wheeler et al., showed that this variant was **not** associated with fasting glucose [3]. Unfortunately, for various technical reasons, GWASs have generally failed to include analysis of variants on the X chromosome [6]. Despite this, 2 previous GWASs of numerous red cell measures in African Americans fortunately did analyze variants on the X chromosome. They showed that the minor allele (T) at rs1050828 was associated with numerous red cell parameters: lower hematocrit, hemoglobin, red cell count, and Red cell Distribution Width (RDW); higher mean corpuscular volume and mean corpuscular hemoglobin concentration [7,8]; as well as principal component 2, formed by multivariate red cell measures [8]. The combined effect of the variant on multiple red cell parameters, along with its lack of association with fasting glucose, led the authors to conclude that its association with HbA1c is through erythrocytic mechanisms, likely through reducing red cell lifespan. However, red cell lifespan is difficult to measure in large cohorts. Likely related, a GWAS of serum bilirubin levels in Sardinians identified variants at G6PD to be associated [9]. Arguing for a causal role of G6PD, the genetic effect was attenuated when G6PD enzyme activity was included in the model. Although G6PD enzyme activity has been shown to be highly heritable [10] with, as expected, strong evidence for X-linked contribution, there has not yet been a GWAS. Unfortunately it appears that G6PD enzyme activity was not measured in any of the African American cohorts studied [3], nor were reticulocyte counts available.

## Shift from glucose to HbA1c to diagnose type 2 diabetes: rs1050828 results in approximately 650,000 cases of undiagnosed type 2 diabetes among African Americans

Just like hypertension and blood pressure, the diagnosis of type 2 diabetes (T2D) is just a threshold on a quantitative trait: the threshold has changed over time as data regarding the risk for long-term complications at various levels of glucose/HbA1c has been more accurately determined using long-term longitudinal epidemiologic studies. The reason that this G6PD variant has potential clinical importance is that, in recent years, many countries have changed recommendations for the diagnosis of T2D to HbA1c from either fasting glucose or oral glucose tolerance tests for a combination of reasons [11,12]. The implication of the genetic association is that hemizygous T males at this variant would need to have an uncorrected HbA1c approximately 0.8% higher (i.e., ≥7.3% [56 mmol/mol] as opposed to ≥6.5% [48 mmol/mol]) than someone without the variant to meet criteria for diabetes diagnosis. If HbA1c were the only test used to diagnose T2D, then it would be diagnosed in such individuals longer after disease onset compared to those without the variant. This would expose them to a longer undiagnosed period while having higher glycemia: these 2 facts would be expected to place them at increased risk for long-term diabetes complications—major causes of morbidity and mortality for people with T2D. Such variants could contribute to the higher risk for long-term diabetes complications in African Americans than European Americans [13]. There would be similar implications for the identification of prediabetes.

Compared to the conventional criteria for T2D of HbA1c ≥ 6.5% (48 mmol/mol), the authors argue that sex- and genotype-specific thresholds should be used to diagnose T2D: ≥5.7% (39 mmol/mol) for T males, ≥5.8% (40 mmol/mol) for TT females, and ≥6.2% (44 mmol/mol) for CT females. Wheeler et al., estimate that approximately 650,000 African Americans (2%, out of an estimated population of about 30 million) would have their T2D undiagnosed because of this single genetic variant (approximately 430,000 males and 180,000 females) if HbA1c were the only test used for T2D diagnosis. For the estimation, they assumed that the variant has an allele frequency of 11% and is in Hardy–Weinberg equilibrium (in females). Geographic heterogeneity in the proportion of African ancestry in African Americans [14,15] would be expected to vary the variant frequency across the United States and could therefore alter the number of individuals affected. Given that, worldwide, approximately 400 million people are G6PD deficient [16], approaching the global prevalence of T2D, then the global health impact of variants at G6PD on T2D diagnosis using HbA1c could be staggering. The vast majority of people with G6PD deficiency are undiagnosed, and screening is not routine [16].

## Population genetics of rs1050828

Data from the 1000 Genomes Project provide allele and genotype frequencies for rs1050828 across a number of different populations. In the African populations, minor allele frequency is as follows: African Caribbeans in Barbados (ACB): 13%; Americans of African Ancestry in south-west USA (ASW): 17%; Esan in Nigeria (ESN): 16%; Mende in Sierra Leone (MSL): 7%; Gambian in Western Divisions in the Gambia (GWD): 4%; Luhya in Webuye, Kenya (LWK): 18%; Yoruba in Ibadan, Nigeria (YRI): 21% [17]—indicating that this variant is common in many African populations. In addition, allele frequency is 2% in both Colombian in Medellin, Columbia (CLM) and Puerto Ricans in Puerto Rico (PUR).

## Other G6PD alleles and other loci

Partly because of the base reference panel that Wheeler used (HapMap phase 2 [18]), there were only 30 mother-father-adult child African trios (from the Yoruba in Ibadan, Nigeria), resulting in 90 X chromosomes. The number of rs1050828 variant alleles in the chromosomes was only 13. The number of other G6PD variants that are known to cause G6PD deficiency that were imputed in their study would be small, making imputation of these variants challenging. However, 2 major improvements have occurred since the analysis plan that Wheeler used was implemented [3]: the 1000 Genomes Project [17] and the Haplotype Reference Consortium [19]. Both consortia have provided genetic data from genotyping and/or sequencing of larger numbers of individuals from more diverse ethnic origins. The use of these as references will likely increase the number of independent variants associated with HbA1c, not only at G6PD but also at other loci. For example, the sickle cell trait (rs334) has recently been associated with lower HbA1c in African Americans based on an expanded number of subjects from 2 of the cohorts included in the Wheeler paper [20,21]. Future challenges at G6PD locus will involve describing the sex- and genotype-specific effects of each variant on HbA1c, since we cannot assume that other G6PD alleles have the same magnitude of effect—some may be larger or smaller, requiring genotype-dependent adjustment of HbA1c thresholds. At G6PD in females, such data will enable the analysis of multiple loci within the locus. For example, some women may be compound heterozygotes for 2 different variants that cause G6PD deficiency, 1 each on their maternal and paternal X chromosomes. Depending on which X chromosome is inactive in red cell precursors, this could have an important influence on HbA1c.

## X chromosome inactivation

Larger variance in HbA1c in heterozygous (CT) than in homozygotes females is an interesting observation from this study (CC, Fig 3 [3]). It could be a result of smaller heterozygote sample size but could also indicate unidentified interacting factor(s). Such factors could include X-inactivation and other ungenotyped and/or poorly imputed variants on the other parental haplotype. G6PD is subject to X-inactivation, but X-inactivation status was not determined in heterozygous females in any of the studies included [3]. Skewed X-inactivation typically increases with age, so the genetic effect on HbA1c could become larger with age in heterozygous females. Depending on which X chromosome is inactive in red cell precursors of heterozygous females, this could have an important clinical application since the effect on HbA1c could either be minimal or similar to that seen in female T/T homozygotes.

## Ethnic differences in HbA1c

Since HbA1c was introduced for T2D diagnosis, heated discussion has ensued regarding whether ethnic differences in HbA1c have implications for both T2D diagnosis as well as glycemic control in people with diabetes [22,23]. Genetic variants such as rs10508282 could play a role but, by itself, cannot explain the **higher** HbA1c in African Americans than in European Americans, since the effect of the variant allele (which is mostly absent in Europeans) is to **lower** rather than raise HbA1c. Alternatives for T2D diagnosis have considered other glycated proteins including glycated serum albumin or fructosamine, either alone or in combination with HbA1c. However, as has been commented recently, no clinical trials have used alternative glycated proteins to link them to risk of long-term diabetes complications, and standardization of these assays has not yet been achieved [24].

## Implications for glycemic control in people with diabetes

Although it was not the focus of Wheeler and colleagues [3], there are also potential implications for people with diabetes. HbA1c is the most common laboratory test performed in people with diabetes, used to measure recent glycemia, and has been the target of many diabetes interventions. Discrepancies between self-monitored glucose and HbA1c are often seen in people with diabetes. Treatment decisions, including escalation of medical therapy in people with T2D are determined, in part, based on HbA1c. Although it has not been directly shown, it is reasonable to assume that the variant has a similar effect on HbA1c in people with diabetes. Previously, it has been shown that males with type 1 diabetes (T1D) and Mediterranean G6PD deficiency have approximately 1% lower HbA1c compared to those without G6PD deficiency [25,26]. Importantly, retinopathy was present only in those males with T1D with G6PD deficiency but not in those without G6PD deficiency, despite similar diabetes duration [26]. Similar results have been observed in other populations [27]. (NB: likely these HbA1c were not standardized). In parallel, more recent data from clinical studies that used repeated glucose measures from Continuous Glucose Measurements along with frequent contemporaneous HbA1c from the same individuals with diabetes showed that there is between-individual heterogeneity in the relationship between these 2 measures [28]. Genetic differences are prime candidates to explain much of this variation. Finally, genetic factors may also contribute to heterogeneous benefits and harms of intensive therapy in people with T2D, as has been observed in some clinical trials [29].

## Take home message and future directions

The G6PD variant rs1050828 is common in people of African and African American origin: it has a large effect on HbA1c, which, if ignored, can result in people failing to meet criteria for T2D using HbA1c while being classified as having T2D using glucose tests. National clinical practice guidelines need to be revisited. Individuals with this variant should either be screened for T2D using glucose, or sex-and genotype-adjusted thresholds for HbA1c should be used. There are also likely implications for the measurement of glycemic control using HbA1c in people with diabetes. Subjects of European ancestry have been the disproportionate focus of GWASs; as the costs for genotyping have dropped recently, larger sample sizes of much more diverse individuals of non-European ancestry are needed to identify other loci that have clinical relevance.

## Abbreviations

ACB: African Caribbeans in Barbados
ASW: Americans of African Ancestry in southwest USA
CLM: Colombian in Medellin, Columbia
ESN: Esan in Nigeria
G6PD: Glucose-6-phosphate dehydrogenase
GWAS: genome-wide association study
GWD: Gambian in Western Divisions in the Gambia
LWK: Luhya in Webuye, Kenya
MSL: Mende in Sierra Leone
PUR: Puerto Ricans in Puerto Rico
RDW: Red cell Distribution Width
SNP: single nucleotide polymorphism
T1D: type 1 diabetes
T2D: type 2 diabetes
YRI: Yoruba in Ibadan, Nigeria
